# Supplementary material for: Validity and reliability of the German translation of the Diabetes Foot Self-Care Behavior Scale (DFSBS-D)
Source: PLoS One. 2022 Jun 3;17(6):e0269395. doi: 10.1371/journal.pone.0269395 (PMC9165872; doi:10.1371/journal.pone.0269395)
Supplement: S1 File — (PDF) [file pone.0269395.s001.pdf]

# The Diabetes Foot Self-Care Behavior Scale – German Version (DFSBS-D)

**Teil 1 - Die untenstehenden Fragen beziehen sich auf die Pflege Ihrer Füße in den letzten 7 Tagen. Wenn Sie in den letzten 7 Tagen krank waren, antworten Sie bitte in Bezug auf die letzten 7 Tage, an denen Sie nicht krank waren.**

| | Anzahl der Tage, an denen die Tätigkeit in den letzten 7 Tagen ausgeführt wurde. | | | | | | | |
|---|---|---|---|---|---|---|---|---|
| 1. Ich untersuche (mein/e Pfleger/in untersucht) meine Fußsohlen. | 0 | 1 | 2 | 3 | 4 | 5 | 6 | 7 |
| 2. Ich untersuche (mein/e Pfleger/in untersucht) meine Zehenzwischenräume. | 0 | 1 | 2 | 3 | 4 | 5 | 6 | 7 |
| 3. Ich wasche (mein/e Pfleger/in wäscht) meine Zehenzwischenräume. | 0 | 1 | 2 | 3 | 4 | 5 | 6 | 7 |
| 4. Ich trockne (mein/e Pfleger/in trocknet) nach dem Waschen meine Zehenzwischenräume. | 0 | 1 | 2 | 3 | 4 | 5 | 6 | 7 |

**Teil 2 - Bitte markieren Sie die Antwortmöglichkeit, die am ehesten Ihrem Verhalten entspricht.**

| | Nie | Selten | Manchmal | Häufig | Immer |
|---|---|---|---|---|---|
| 1. Wenn meine Haut trocken ist, versorge ich (versorgt mein/e Pfleger/in) meine Füße mit einer Feuchtigkeitscreme. | 1 | 2 | 3 | 4 | 5 |
| 2. Bevor ich meine Schuhe anziehe, überprüfe ich (überprüft mein/e Pfleger/in) die Innenseite der Schuhe. | 1 | 2 | 3 | 4 | 5 |
| 3. Ich laufe neue Schuhe langsam ein. | 1 | 2 | 3 | 4 | 5 |
